# Supplementary figures and images for: Dystrophin involvement in peripheral circadian SRF signalling
Source: Life Sci Alliance. 2021 Aug 13;4(10):e202101014. doi: 10.26508/lsa.202101014 (PMC8363758; doi:10.26508/lsa.202101014)

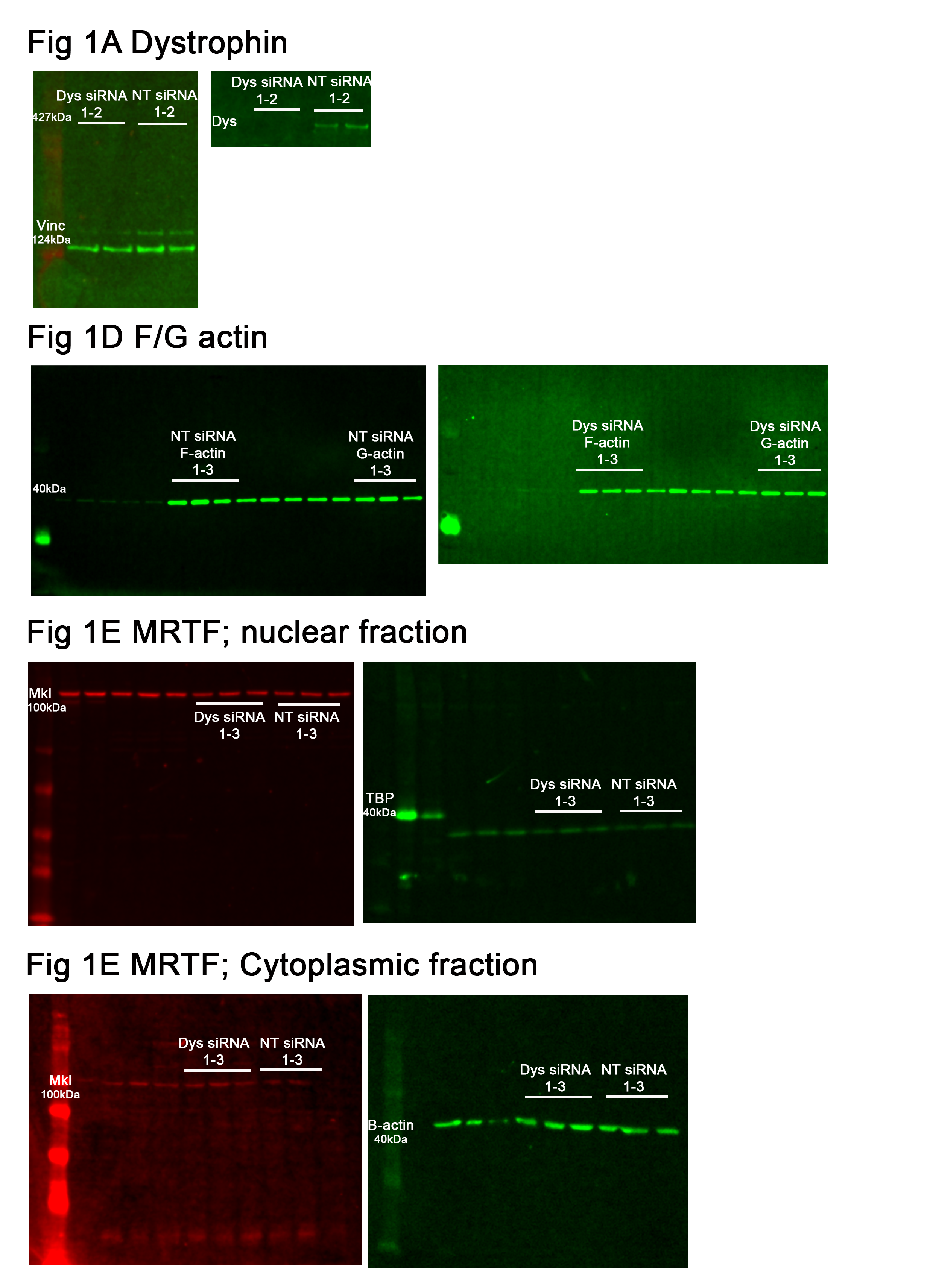

Supplement: Supplementary file 1 [file LSA-2021-01014_SdataF1.tif]

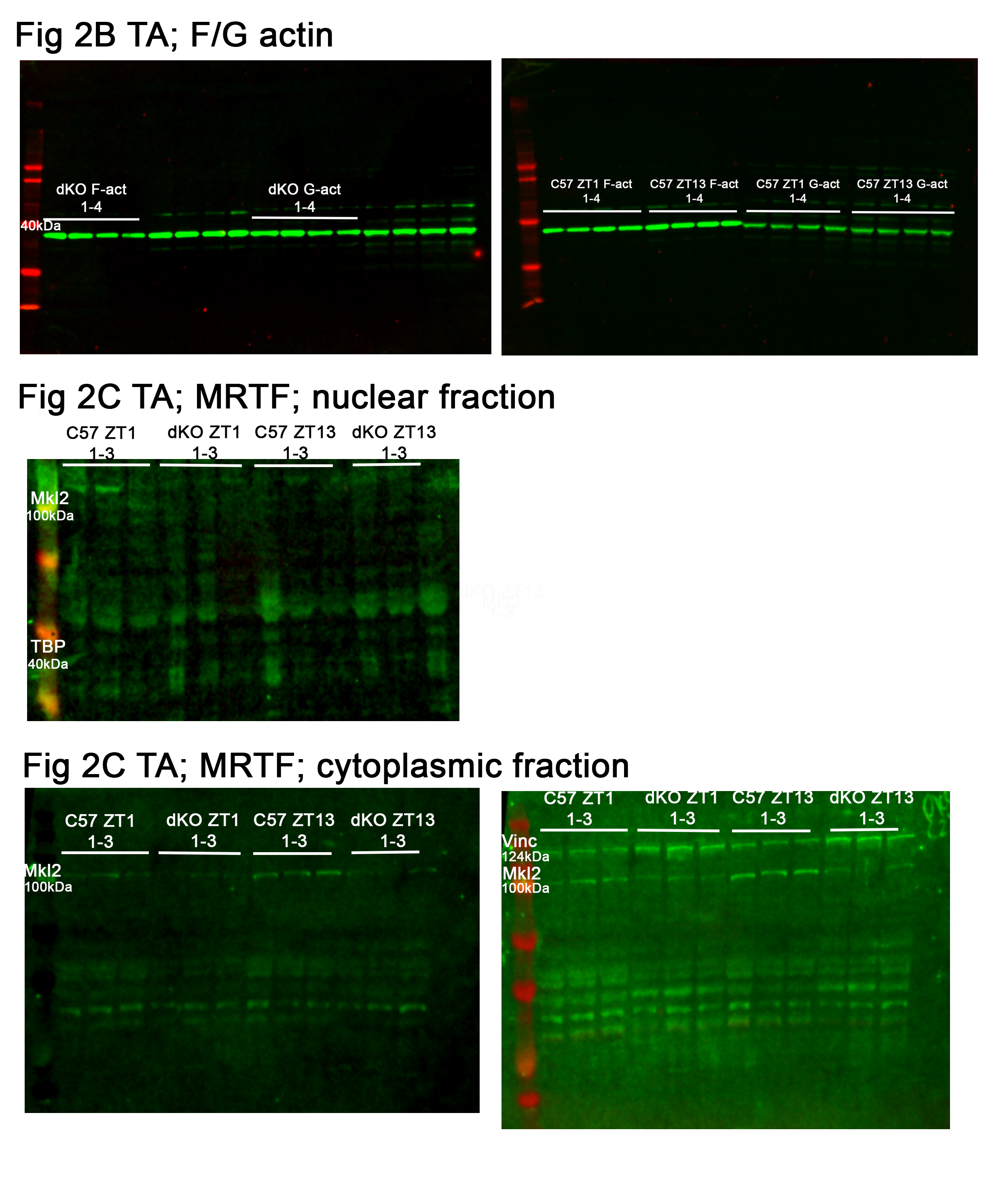

Supplement: Supplementary file 2 [file LSA-2021-01014_SdataF2.tif]

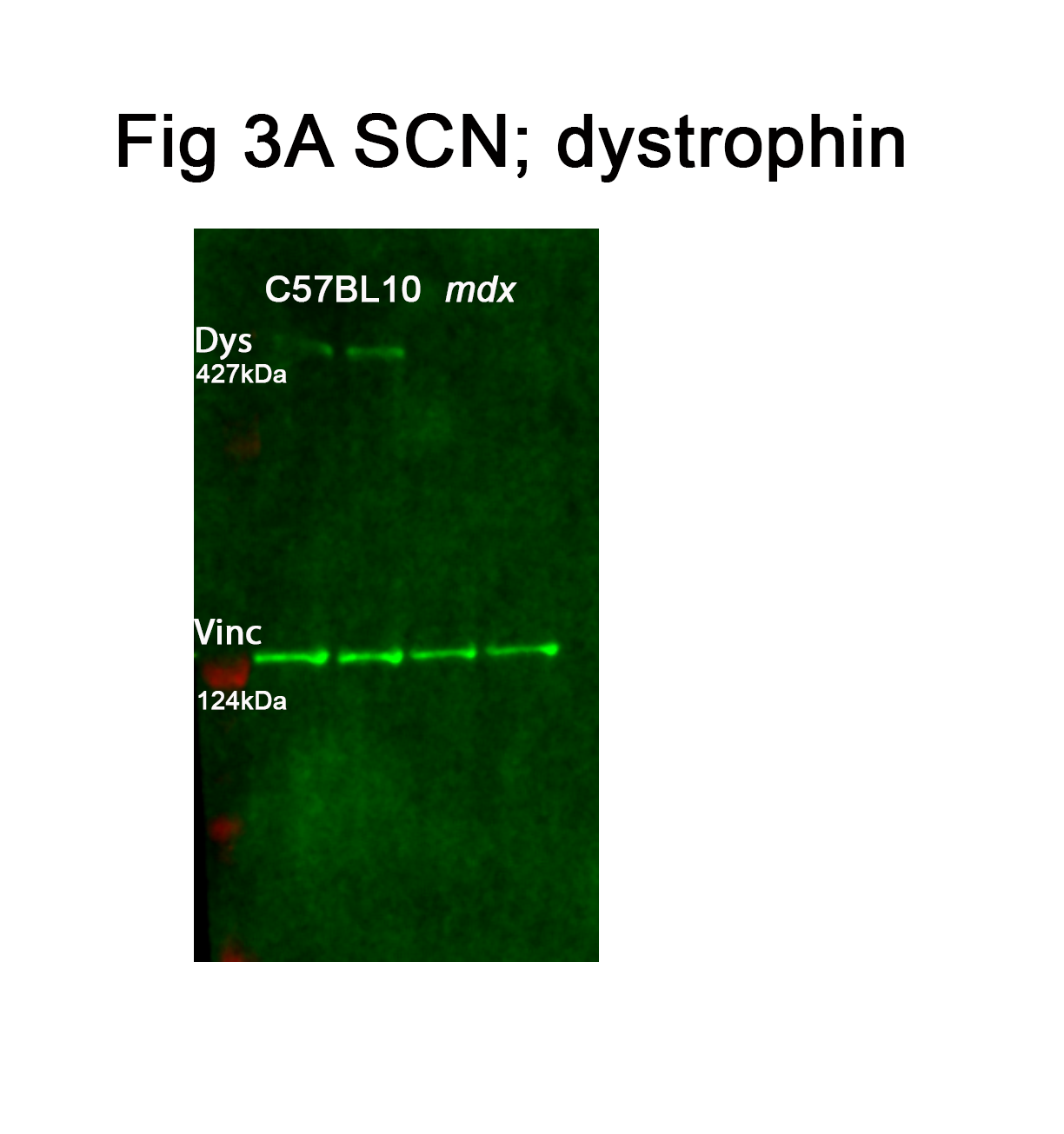

Supplement: Supplementary file 3 [file LSA-2021-01014_SdataF3.tif]

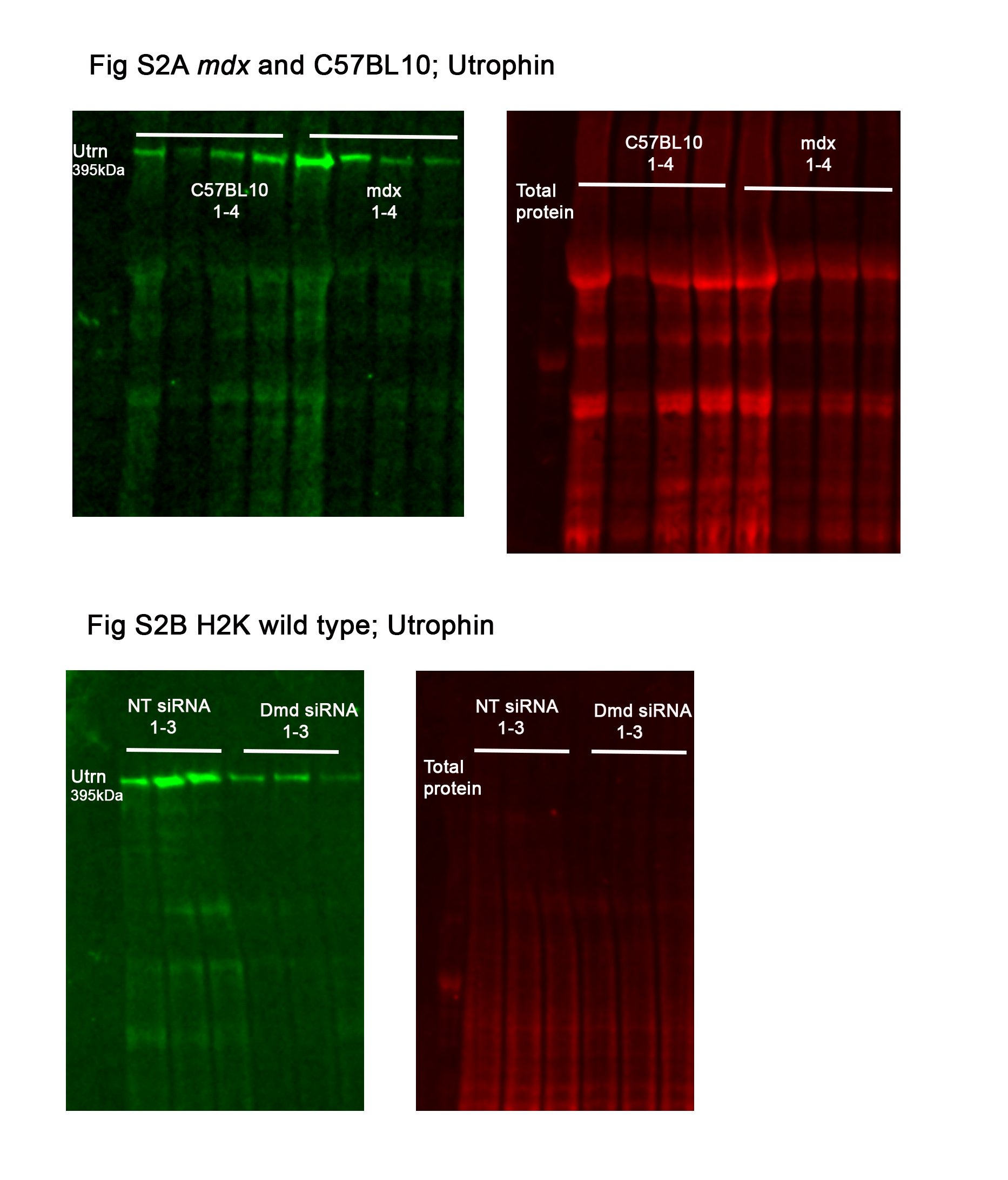

Supplement: Supplementary file 4 [file LSA-2021-01014_SdataFS2.tif]
